# Supplementary material for: Population-based epidemiological analysis of acute pyelonephritis and antibiotic prescription in Spain (2009–2018)
Source: PLoS One. 2025 Dec 19;20(12):e0338447. doi: 10.1371/journal.pone.0338447 (PMC12716755; doi:10.1371/journal.pone.0338447)
Supplement: S2 File — (DOCX) [file pone.0338447.s002.docx]

**Supplement 2. Antibiotic groups and active principles prescribed for acute pyelonephritis**

| Antibiotics group | Principios activos* |
| --- | --- |
| Cephalosporins |  |
| Cefalosporinas de 1ª (0.02%)  Cefalosporinas de 2ª (17.7%)  Cafalosporinas de 3ª (21%)  Cefalosporinas de 4ª (0 %) | Cephalexin (0.02%), cefazolin (0.003%), cefadroxil (0.003%), cefoxitin (0%), cephalothin (0%)  Cefuroxime (17.7%), cefaclor (0.01%)  Cefixime (15.2%), cefditorene (5.4%), ceftibuten (0.3%), ceftriaxone (0.08%), cefpodoxime (0.03%) , cefotaxime (0%), ceftazidime (0%)  Cefepime (0%) |
| Quinolones | Ciprofloxacin (22.7%), levofloxacin (4.5%), norfloxacin (2.3 %), ofloxacin (0.3%), moxifloxacin (0.2%), ác pipemidico (0.02%), ác nalidixico (0%) |
| Penicillin_combined | Amoxi-clavulanic (21.4%), amoxicillin (0.8%), ampicillin (0.07%) |
| Fosfomycin | Fosfomycin (6.8%) |
| TMP/SMX | Sulfamethoxazole y trimethoprim (0.8%) |
| Aminoglycosides | Tobramycin (0.5%), gentamicin (0.2%) |
| Macrolides | Azithromycin (0.2%), clarithromycin, (0.08%, erythromycin (0.05%), spiramycin (0.003%) |
| Nitrofurantoin | Nitrofurantoin (0.4%) |

* Percentage of use over the total prescribed antibiotics
